# Conversion of hulled into naked barley by Cas endonuclease-mediated knockout of the *NUD* gene

**DOI:** 10.1186/s12870-020-02454-9

**Published:** 2020-10-14

**Authors:** Sophia V. Gerasimova, Christian Hertig, Anna M. Korotkova, Ekaterina V. Kolosovskaya, Ingrid Otto, Stefan Hiekel, Alex V. Kochetov, Elena K. Khlestkina, Jochen Kumlehn

**Affiliations:** 1 Institute of Cytology and Genetics, SB RAS, Novosibirsk, 630090 Russia; 2 Novosibirsk State University, Novosibirsk, 630090 Russia; 3 Kurchatov Genomics Center, Institute of Cytology and Genetics, SB RAS, Novosibirsk, 630090 Russia; 4 Leibniz Institute of Plant Genetics and Crop Plant Research (IPK), 06466 Gatersleben, Germany; 5 Vavilov Institute of Plant Genetic Resources (VIR), Saint Petersburg, 190000 Russia

**Keywords:** *Hordeum vulgare*, Naked barley, Cas9, Gene knockout, Domestication, Protoplasts, Targeted mutagenesis

## Abstract

**Background:**

The naked caryopsis character in barley is a domestication-associated trait defined by loss-of-function of the *NUD* gene. The functional *NUD* gene encodes an Apetala 2/Ethylene-Response Factor (AP2/ERF) controlling the formation of a cementing layer between pericarp and both lemma and palea. The downstream genes regulated by the NUD transcription factor and molecular mechanism of a cementing layer formation are still not sufficiently described. A naturally occurring 17-kb deletion in the *nud* locus is associated with the emergence of naked barley. Naked barley has been traditionally used for food and nowadays is considered as a dietary component for functional nutrition.

**Results:**

In the present study, we demonstrate that targeted knockout of the *NUD* gene using RNA-guided Cas9 endonuclease leads to the phenotype conversion from hulled to naked barley. Using in vivo pre-testing systems, highly effective guide RNAs targeting the first exon of the *NUD* gene were selected. Expression cassettes harboring the *cas9* and guide RNA genes were used to transform barley cv. Golden Promise via *Agrobacterium*-mediated DNA transfer. The recessive naked grain phenotype was observed in 57% of primary transformants, which indicates a frequent occurrence of homozygous or biallelic mutations. T-DNA-free homozygous lines with independently generated mutations in the *NUD* gene were obtained in the T1 generation. At homozygous state, all obtained mutations including one- and two-amino acid losses with the translational reading frame being retained invariably caused the naked grain phenotype.

**Conclusions:**

The hulled and naked barley isogenic lines generated are a perfect experimental model for further studies on pleiotropic consequences of *nud* mutations on overall plant performance under particular consideration of yield-determining traits. Due to the high β-glucan content of its grains, naked barley is considered as being of particular dietary value. The possibility to convert hulled into naked barley cultivars by targeted mutagenesis allows breeders to extend the potential utilization of barley by the provision of functional food.

## Background

One of the most clearly distinguishable traits of barley which emerged during the process of domestication is the naked versus the hulled caryopsis character. Barley cultivation is dated back to about 10,000 years before present day [1]. The naked barley trait occurred early during domestication, that is, already in the 7th millennium B.C. It appeared in different combinations along with other characteristics, such as various grain colors and two- or six-rowed spikes [2]. While barley domestication is well established as being of multiple independent origin, naked grain barley is usually considered to be monophyletic [3, 4]. The vast majority of naked barley accessions are harboring a 17-kb deletion at the same locus located on the long arm of chromosome 7H [4–7]. The locus is called *NUD* (for *nudum*) and in hulled varieties, it contains a gene (*NUD*) encoding an Ethylene Response Factor (ERF) family transcription factor belonging to the group of Wax Inducer 1/Shine 1 (WIN1/SHN1)-like transcription factors [5]. Re-sequencing of the *NUD* locus in large panels of barley varieties showed the intact *NUD* gene in all hulled accessions, whereas all naked ones featured either the aforementioned 17-kb deletion or a T643A amino acid conversion [6]. An analysis of X-ray-induced naked grain mutants confirmed the expectation that the *NUD* gene carried non-synonymous single nucleotide polymorphisms in all cases [5]. More recently, targeted mutagenesis of the *NUD* gene was also demonstrated to cause the appearance of naked grains on primary transgenic plants [8].

Genotypes with naked grains were selected and fixed during domestication of the majority of cereal crops, but the origin of this trait is not uniform. The naked grain of bread wheat is associated with soft glumes in contrast to tough glumes of wild progenitors [9]. The mechanism of hulled grain formation in barley is apparently different from wheat in that it relies on the occurrence of a lipid-based cementing layer between pericarp and hull [5, 10]. The exact mechanism of cementing layer formation and the role of the *NUD* gene in this process are still not well understood. The *NUD* gene was found to be expressed during grain development in the integument that constitutes the coat of the ovule, while the cementing layer occurs at the surface of the pericarpal epidermis [5]. There are no other genes known to be involved in hulled barley control. The recent genome-wide association analysis on 525 spring barley landraces corroborated the key role of the *NUD* gene in naked grain formation [7]. A comparative transcriptome analysis of hulled and hulless barley accessions revealed a suppression of genes involved in pericarpal cuticle organization in hulled barley. It was hypothesized that the *NUD* gene may be a master regulator of these genes to increase cuticle permeability thereby causing the hull–caryopsis fusion [11]. However, under consideration of the different genetic backgrounds of the compared germplasm, the observed differences could not be directly related to the *NUD* gene. Consequently, thus far available data do not allow for an elucidation of the molecular mechanisms of cementing layer formation in hulled barley.

The use of customizable endonucleases allows one to induce desirable modifications at defined genomic loci [12]. With the emergence of RNA-guided Cas endonucleases, the number of examples of site-directed genome modification has been strongly increasing in crop plants [13]. The technology is currently well established in barley, including both reverse genetics studies and improvement of agronomically important characteristics [14].

The present study aims to strengthen the body of evidence for the causal effect of loss-of-function of the *NUD* gene on the formation of non-adherent hulls in barley. This is demonstrated by Cas endonuclease-mediated generation and comparison of isogenic lines carrying either functional or non-functional alleles of the *NUD* gene in the context of an identical, transgene-free genetic background. A further goal is the generation of such perfectly isogenic lines which will facilitate future studies on anticipated pleiotropic effects of the naked barley character on qualitative and quantitative plant features. And finally, the conversion of hulled into naked barley without any linkage drag shall provide breeders with the unprecedented opportunity of readily introducing performance-related features of the most advanced hulled barley germplasm to naked barley.

## Results

### Targeted knockout of the *NUD* gene

**Fig. 1:**
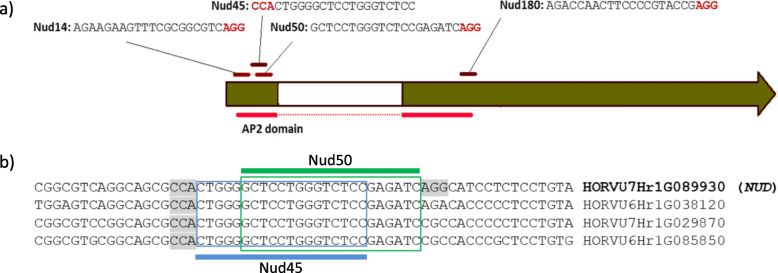
**a** Target motifs selected in the coding sequence of the *NUD* gene. Protospacer-adjacent motifs (PAM) are indicated by red letters. **b** Target motifs for selected gRNAs. The *NUD* gene code is marked bold, the Nud45 target motif is marked by a blue frame, the Nud50 target motif by a green frame, and the PAM (NGG) is indicated in grey

Four different target motifs were selected within the Apetala 2 domain-encoding region of the *NUD* gene (Fig. 1a). For these sites, guide RNAs (gRNAs) were designed (Additional file 1, Table S1) and incorporated in the pSH121 generic vector [15].

**Fig. 2:**
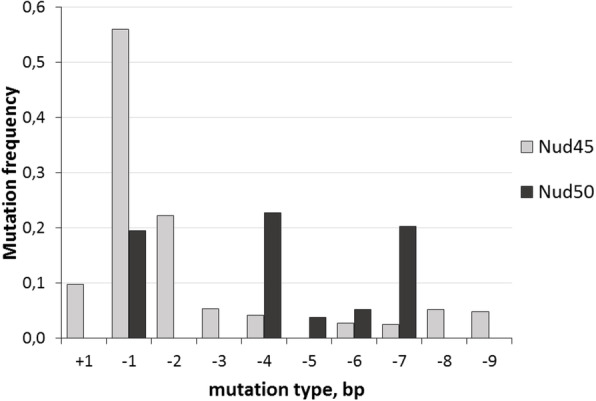
Frequency and pattern of target-specific mutations in transfected protoplasts

Two different in vivo test systems were applied to select the appropriate gRNAs for stable plant transformation. For the preliminary selection of gRNAs, a previously described test system based on reporter gene restoration upon targeted mutagenesis of test constructs was used [16]. Co-bombardment of barley epidermis with pairs of a *cas9*/gRNA and a corresponding TARGET vector followed by quantification of mutagenesis efficacy showed that all designed gRNAs were capable of inducing mutations in their cognate target motifs in vivo. The activity, being measured as the ratio of cells showing reporter restoration per total transgene-expressing cells, ranged from 0.27 to 0.53 (Additional file 1, Table S1). Based upon these results, it was decided to select two gRNAs, namely Nud45 and Nud50, for further experiments. Both corresponding target motifs are located within the first exon of the *NUD* gene in a highly conserved region of the *AP2*-domain (Fig. 1a), which provides the highest probability to induce loss-of-function mutations. To reveal the in situ mutation frequencies and patterns induced by the preselected gRNAs in the *NUD* gene, an assay was utilized, that relies on transient expression in protoplasts. To this end, transformation vectors carrying the Nud45 and Nud50 gRNAs were introduced into barley mesophyll protoplasts through polyethylene glycol-mediated transfection. Genomic DNA extracted from transformed protoplasts was used to determine the efficiency of target-specific mutagenesis. After normalization to *GFP* co-transformation efficiency, deep-sequencing of amplicons from the target regions revealed high portions of mutated reads for both *cas9*/gRNA vectors tested; 100% for Nud45 and 71% for Nud50 (Additional files 2 and 3, Tables S2, S3). The frequency and pattern of mutations induced within the target motifs appeared to be significantly different (*p* < 0,01) between two gRNAs, according to the two-sample Kolmogorov-Smirnov test. The distribution of mutation types obtained is presented in Fig. 2.

The Nud45 gRNA showed the highest activity in both preliminary tests. The off-target analysis revealed four predicted identical target motifs for the Nud45 gRNA in the barley genome which are located at different genomic loci including the *NUD* gene. Being located close to the Nud45 target motif, the on-target of the Nud50 gRNA also has three entirely matching 20-nt off-target counterparts in the genome, but none of these is followed by NGG, that is, the protospacer-adjacent motif (PAM) required for Cas9 to bind and process the target (Fig. 1b). Both of the selected gRNAs were used for stable plant transformation, which enabled us to compare their activity between transient expression assay and stably transformed plants as well as to evaluate the off-target effects in perfectly matching off-target loci with versus without functional PAM.

**Fig. 3:**
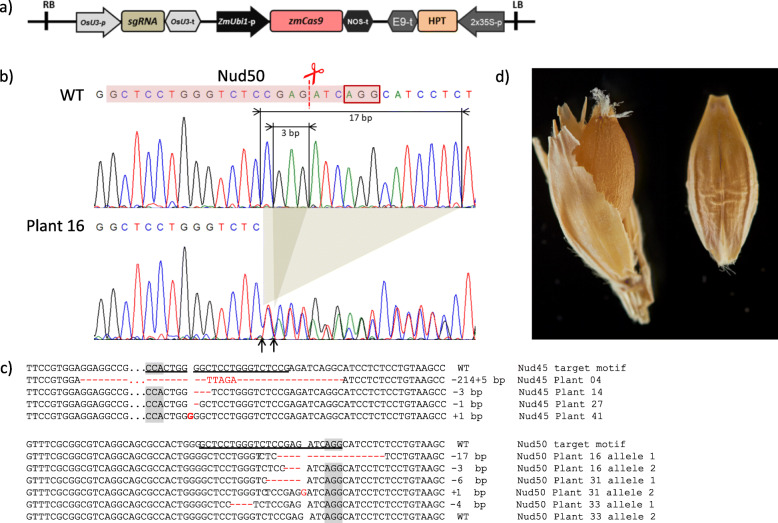
**a** Binary vector T-DNA architecture, OsU3-p – rice U3 promoter; gRNA – chimeric guide RNA; OsU3-t – rice U3 terminator; ZmUbi1-p – maize *Ubiquitin 1* promoter; zCas9 – maize codon-optimized *Streptococcus pyogenes cas9* endonuclease gene; Nos-t – 3′-signal of *Agrobacterium tumefaciens nopaline synthase* gene; E9-t – pea *Ribulose-1,5-Bisphosphate Carboxylase Small Subunit* (rbcS) E9; HPT – *hygromycin phosphotransferase*, 2x35S-p – doubled enhanced CaMV 35S promoter. **b** Mutation detection in T0, Sanger chromatogram of intact (WT) and mutated (plant 16) Nud50 target motif. The sequence with colored background indicates the target motif with the PAM being also marked by a red frame. Arrows indicate fragments deleted from the WT sequence and the corresponding ligation points in the mutant alleles of the biallelic plant 16. **c** Examples of mutated alleles of the *NUD* gene found in T0 population after mutagenesis directed by the Nud45 and Nud50 gRNAs. Target motifs are underlined, the PAM is marked grey. **d** Phenotype of grain harvested from mutant plant (left) in comparison to wild-type ‘Golden Promise’ grain (right)

Next, *cas9* and gRNA expression units were transferred from the plasmids used for the protoplast assay into binary vectors for *Agrobacterium*-mediated plant transformation (Fig. 3a). After DNA transfer to immature embryos, 46 independent primary transgenic plants (T0) were obtained for the Nud45 gRNA and 48 transformants for the Nud50 gRNA. The analysis of Sanger sequencing chromatograms of leaf samples taken from individual T0 plants (Fig. 3b) revealed that 35 plants carried mutations in the Nud45 and 20 in the Nud50 target. The detected mutations were either homozygous (where no wild-type and no more than one mutant allele was found), heterozygous (where a single mutation was combined with the wild-type sequence), or biallelic (where two different mutant alleles were present). Of note, heterozygous and biallelic mutants cannot be discriminated from chimeric ones which carry different sectors with wild-type and/or various mutant alleles. Also owing to possible chimerism, the leaf samples taken were not necessarily representative for the whole plants. The leaf samples taken from 8 Nud45 primary transgenic plants did not contain more than one mutant *NUD* allele each; 5 plants carried a 1 bp deletion, 1 plant a 3 bp deletion, 1 plant a 1 bp insertion and 1 plant a ~ 200 bp deletion combined with a 5 bp insertion. All detected mutations in Nud50 primary mutants were in heterozygous or biallelic state (Fig. 3c). However, only few analyzed plants produced naked grains (Fig. 3d), others formed hulled or both hulled and naked caryopses, suggesting a sectorial presence of wild-type, homozygous, biallelic and heterozygous alleles in chimeric primary mutant plants (Table 1).

**Table 1:** Analysis of leaf samples taken from T0 plants with *cas9*/gRNA expression units being stably integrated

gRNA	Genotype				Phenotype			
	transgenic T0 plants	mutated plants	homozygous mutations	Efficiency (%)	Plants with grains	only hulled grains	hulled and naked grains	only naked grains
Nud45	46	35	8	76	40	7	27	6
Nud50	48	20	0	42	43	22	16	5

The off-target analysis was performed only for T0 mutants with detected homozygous mutations. Seven T0 plants from the Nud45 mutant population harboring different types of homozygous mutations in the *NUD* gene were checked for the presence of mutations in the three perfectly matching off-target loci. As a result, mutations were detected at all three off-target loci in every on-target mutant plant.

### Generation of T-DNA-free knockout lines

In order to develop T-DNA-free mutant lines, three plants having different mutation types were selected from the Nud45 and Nud50 T0 populations for further propagation and analysis (Fig. 3c, Additional file 4, Table S4). Five T1 grains (grown on T0 plants) were planted per selected primary Nud45 mutant and 30 T1 grains per Nud50 T0 mutant.

**Fig. 4:**
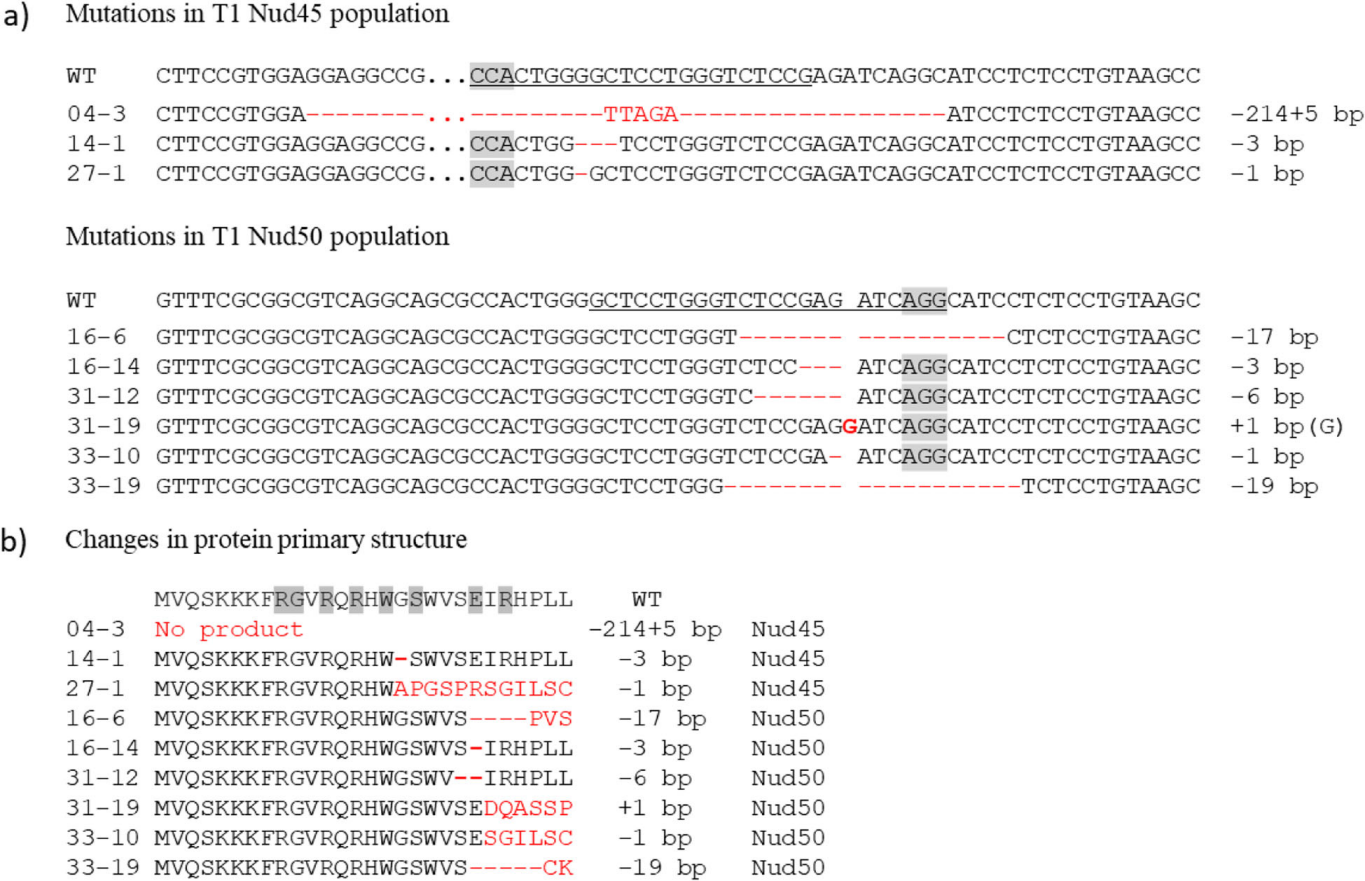
Cas9/gRNA-induced homozygous mutations in the *NUD* gene found in the T1 mutant population. Numbers correspond to individual plants. **a** Alterations in the nucleotide sequence, the PAM is marked grey. **b** Alterations in the amino acid sequence of the first exon. The DNA-binding motif is marked grey

**Fig. 5:**
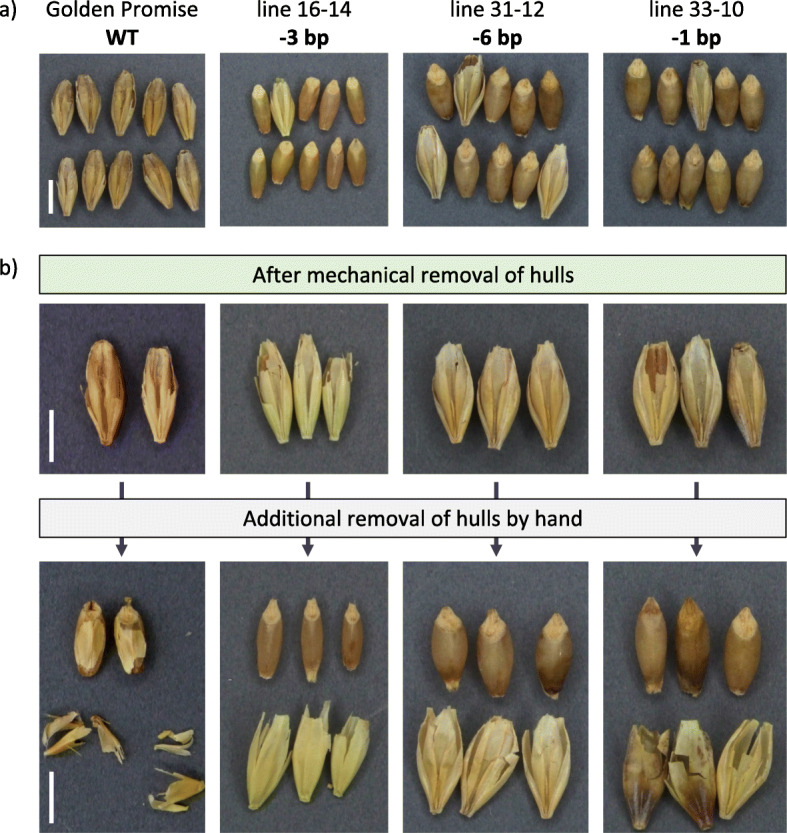
T2 grains collected from *NUD* homozygous knockout Nud50 T1 mutants. **a** Grain samples collected from individual plants from three independent mutant lines (No. 16, 31 and 33) after mechanical threshing. **b** Manual removal of hulls that remained attached to grains after the standard threshing procedure. White bars represent 5 mm

In the Nud45 T1 families, two transgene-free plants having homozygous mutations in the *NUD* gene were identified. Both of these, however, also carried homozygous mutations in all three perfectly matching off-target loci. All other T1 plants from this population had inherited the *cas9* and gRNA transgenes along with mutations in all four investigated target loci. In the Nud50 T1 families, transgene-free segregants each harboring one of the following homozygous mutations in the *NUD* gene were identified: 1 bp insertion and three deletions of different length (− 1, − 3 and − 17 bp). In addition, two further types of homozygous deletions (− 6 and − 19 bp) were identified among those Nud50 T1 segregants that still proved transgenic. Among the different mutation patterns found at the on-target sites in both mutant populations, three types of in-frame mutations were identified; single amino acid deletions ΔG17 and ΔE22 as well as a deletion of two amino acids in positions 21 and 22 (ΔSE21/22). The mutations obtained in T1 are shown in Table 2 and Fig. 4. A summary of *NUD* gene mutation types detected in selected primary transgenic plants and corresponding T1 progeny is given in Additional file 4 (Table S4). The naked caryopsis phenotype was present in all plants harboring homozygous mutations in the *NUD* gene including those mutations with the reading frame being retained. Very few individual grains of *NUD* KO homozygous plants remained with hulls after standard mechanical threshing. Yet, their hulls could readily be removed manually. By contrast, the wild-type *NUD* allele in progeny of primary transgenic plants was invariably associated with the formation of hulled grains, i.e. the hulls were neither removable by mechanical threshing nor by hand in all registered cases (Fig. 5).

**Table 2:** Types of homozygous mutations identified in the T1 generation

gRNA	Type of mutation	No. of plants	No. of transgenic plants / No. of transgene-free plants	*NUD* CDS reading frame
Nud45	−1 bp	2	2/0	Shifted
	−3 bp	2	2/0	Retained
	− 214/+ 5 bp	2	0/2	Shifted
Nud50	−1 bp	8	6/2	Shifted
	−3 bp	6	4/2	Retained
	−6 bp	5	5/0	Retained
	−17 bp	8	6/2	Shifted
	−19 bp	1	1/0	Shifted
	+ 1 bp (G)	1	0/1	Shifted

In addition to the three perfectly matching Nud45 off-targets, there are three aforementioned potential off-target sites in the barley genome which are perfectly coinciding with the target-specific part of the Nud50 gRNA, while not having an appropriate PAM site. In all on-target T1 mutants, no mutations were detected whatsoever in these PAM-less off-targets.

## Discussion

### Mutations induced in the *NUD* gene

The target site-specific activity of gRNA-navigated Cas9 and the resultant mutation patterns are highly variable. The validation of gRNA activity in vivo prior to whole-plant genome modification allows one to achieve a high portion of plants with specific alterations in the target motif [17]. Here, two different test systems were used to select efficient guide RNAs for targeted mutagenesis of the *NUD* gene in barley. The results of preliminary tests were in good agreement with mutation frequency and pattern in transgenic plants and their progeny. In this study, different mutation patterns were seen in the two addressed target motifs nud45 and nud50. In the protoplast system, the predominantly occurring mutation induced by the Nud45 gRNA was a one-nucleotide deletion. This proved also to hold true for the T0 plants carrying the same gRNA, where 5 out of 8 homozygous mutants had the very same modification. By contrast, the Nud50 target motif showed more diverse mutation types both in protoplasts and mutant plants. There was a high portion of T0 mutants having both alleles mutated in different, that is biallelic, manner, whereas no homozygous mutants were obtained in these cases. The achieved mutation frequencies for the Nud45 target motif were higher than for the Nud50 target motif in all experiments. In T0 plants, the corresponding frequencies were as high as 76 and 42%, respectively. This was sufficient to obtain the desired recessive phenotype at reasonably high proportion already among T0 plants, from which transgene-free homozygous T1 mutants also efficiently segregated. The high mutation frequency was comparable with results previously reported by Kapusi et al. [18], Holubova et al. [19] and Li et al. [20], where similar principles of target motif selection and gRNA prevalidation had been implemented to knockout the barley *ENGase*, *CKX1* and *Albostrians* genes, respectively.

Two gRNAs used in this study have three perfectly matching 20-nt off-target loci, but in case of the Nud45 gRNA, off-targets are associated with a functional PAM, whereas adjacent to the Nud50 gRNA off-targets there is no appropriate PAM. It is demonstrated that a Nud45 gRNA with high predicted activity is capable of simultaneously inducing mutations at all four different genomic target loci. This opens the opportunity to efficiently knockout gene families using just one or few highly efficient gRNA(s). The Nud45 off-targets are located in *AP2*/*ERF* genes with unknown functions, which are sharing sequence homology with the *NUD* gene. Multiple mutant plants obtained in this study can be further used for reverse genetic studies of those *NUD*-related genes’ function.

### Different mutations in the *NUD* gene lead to the naked grain phenotype

The occurrence of naked grains has previously been observed in primary transgenic barley plants bearing *cas9* and *NUD* gene-specific gRNA expression units [8]. While leaf samples were reported to have homozygous, biallelic or heterozygous/chimeric mutation patterns, there was however no clear association of individual genotypes with phenotypes at the whole-plant level demonstrated. By contrast, in the present study, various *nud* gene mutations detected in T0 plants were shown to be transmitted to transgene-free progeny that were also confirmed not to carry any off-target mutations. Therefore, it was possible to unambiguously assign the naked grain phenotype to homozyogus *nud* mutant segregants. These T1 plants themselves gave rise to offspring invariably carrying naked grains. Not only frameshift mutations but also in-frame mutations associated with the loss of one or two amino acids in the AP2 domain of the *NUD* gene caused the conversion of hulled into naked barley. Both selected target motifs are located within the first exon at the 5′ part of the *AP2* domain. The obtained one- and two-amino acid deletions appear within the nucleotide binding site (Fig. 4b), that is in a highly conserved region. The loss of function associated with these allelic variants, as demonstrated in the present study, provides convergent evidence of the essential role of this domain for the NUD transcription factor.

The hulled caryopsis character is defined by the *NUD* gene expressed in the coat of the ovule thereby regulating the accumulation of cementing substances at the surface of the pericarp. All these tissues are of maternal origin, which means that the hulled or naked caryopsis type solely depends on the mother plant genotype. In some of the T0 plants generated in the present study, the occurrence of both types of grains on the same spike was observed. This phenomenon can be explained by the limited penetrance of Cas9 nuclease activity causing chimeric primary transgenic plants in which induced mutations are confined to some sectors. Some of the *cas9*/gRNA-free T2 grains collected from homozygous T1 mutant plants also had still adherent hulls after standard mechanical threshing, yet in this case the hulls could readily be removed by hand, which was possible owing to the absence of the cementing layer between hull and pericarp.

While previous work on *NUD* function relied on association studies [4–7], the present study adds unambiguous evidence for the causal effect of loss-of-function of the *NUD* gene on the formation of non-adherent hulls in barley. The results obtained also confirm the monogenic control of the caryopsis type in barley and demonstrate the possibility to convert hulled into naked barley by targeted mutagenesis of the *NUD* gene. Furthermore, it was demonstrated that the deletion of no more than a single nucleotide can be sufficient for this conversion.

### Pleiotropic effect of the *nud* locus

Barley has high genetic diversity and comprises multiple combinations of different traits. Consequently, many differences have been observed between naked and hulled barley accessions. However, it is not always clear which of these phenotypes have just coincidentally occurred for instance owing to linkage drag, and which constitute truly causal consequences of a particular *NUD* allele. The most known differences between hulled and naked barley are related to the hull itself. Hulled barley has higher yield and higher crude fiber content. The hull normally constitutes 10–13% of the dry weight of barley grain [21] and the naked barley yields about 88% of hulled barley [22]. In a number of experiments aiming to establish the association between naked grain and other characteristics, contradictory results were obtained. A comparison of two hulled barleys and their naked mutant derivatives revealed only minor differences in yield and growth performance [23]. A further comparison of near-isogenic lines did also not establish that the naked phenotype be associated with any alterations as to amino acid composition of the grain [24], grains per m^2^, grains per spike, plant height, heading date and mildew resistance [22]. Crossings between naked and hulled Himalaya barleys revealed a multigenic nature of their differences [25] and again no evidence for pleiotropic effects of the *nud* locus, and in a similar study it was concluded that allelic variants of the *NUD* locus are unlikely to have an effect on seedling vigour [26]. Likewise, an analysis of doubled haploid lines derived from hybrids of hulled and naked barley did not reveal any pleiotropic effects of the naked phenotype on heading date, maturity, smut resistance, scald resistance, and spike density, but showed associations with lower plant density, reduced plant height as well as lower yield and grain weight [27]. However, the two latter effects may be well explained by the absence of the hull. A reduced number of plants grown per 100 viable grains was shown for naked barley accessions in comparison to hulled ones [28]. While Fusarium head blight severity was shown not to be different in hulled and naked barley, hulled barley accumulated significantly higher amounts of mycotoxins in the grain [29]. Taken together, these data as well as the successful cultivation of naked barley under different regional conditions suggest the absence of deleterious effects of the *nud* gene variant and at worst minor effects on important agronomic characteristics. It has been repeatedly reported that hulless barley has a potential for genetic improvement, and many disadvantages of naked varieties are not directly caused by the *nud* allele and thus may be overcome by site-directed mutagenesis using suitable hulled barley backgrounds.

### Potential of engineered *nud* knock-out lines for the advancement of naked barley breeding and the development of functional food

The presence or absence of the hull is essential regarding the end use of barley grain. Rather than being processed for malting, the naked barley grain had been traditionally used for food in ancient times but was substituted by wheat in many regions including Europe. In the 20th and 21st centuries, the hulled barley has preferentially been cultivated in Europe and used mainly for malting, distilling and animal feed. Strong adhesion of lemma and palea to the pericarp is a major prerequisite of current brewing technologies [10]. However, as an important food source, the hulless barley is cultivated in Asia and northern Africa [30]. Nowadays, the interest in incorporating barley as a dietary component in food products is increasing because of the rising societal demand for food with potential health benefits. Barley contains high amounts of β-glucans. Humans do not have enzymes for β-glucan degradation, which is the reason that these molecules exert their beneficial properties as soluble fibers [31]. It was shown that barley β-glucan consumption is effective for the reduction of blood LDL-cholesterol [32, 33] and reducing visceral fat obesity [34]. The effect of barley β-glucans on reducing the food glycemic index and their short-time blood sugar regulating properties is discussed [35–37]. In several studies, it was shown that naked barley has a higher content of β-glucans [38, 39] and the *nud* locus has been associated with a major QTL for this feature [40]. The effect of *nud* alleles on β-glucan content may be explained either by the closely linked location of the *NUD* gene and genes controlling β-glucan biosynthesis on the 7H chromosome or by a direct effect of the *NUD* gene on cell wall composition in the barley grain. The suppression of cuticle organization genes in hulled barley shown by comparative transcriptome analysis of two barley cultivars [11] and the presence of the cementing layer in hulled barley grain suggest differences in cell wall and cuticle organization in hulled and naked grain. This difference may have an effect on the nutritional value of the grain. The *NUD*/*nud* isogenic lines that are now available can be used to unambiguously confirm or falsify a causal relationship between naked caryopsis type and grain nutrient composition.

To use hulled barley for food, a pearling process is needed. Pearling removes the grain hull along with the surface layer of the grain which is particularly enriched with beneficial nutrients [21]. The use of naked barley for functional food is of particular health value because of optimal nutrient composition and the comparatively low costs of grain processing. The diversity of naked barley has been lost in regions where barley was no longer widely used for food. The introduction of non-adapted naked barley varieties in local breeding programs requires the genetic screening of germplasm collections and phenotyping under field conditions. This process does not allow for the development of dietary products containing naked barley in a short period of time. The possibility to convert current elite material to provide high-performance barley cultivars with naked grains can contribute to the provision of increasingly requested healthy food even beyond those regions where naked barley has been traditionally consumed. The developed and pre-tested highly efficient gRNA structures and genetic constructs for targeted knockout of the *NUD* gene may be directly used to convert advanced hulled germplasm into naked barley for dietary purposes.

## Conclusions

The naked vs. hulled grain characteristic in barley is an example of a trait under monogenic control. The phenomenon of hull adhesion owing to the formation of a cementing layer is peculiar to barley, and there is no evidence in other species regarding WIN1/SHN1-like AP2/ERF factors controlling the formation of hulled or naked grains. The molecular function of the NUD transcription factor is still unknown and is not yet associated with any downstream genes or biochemical pathways. Using Cas endonuclease technology in the present study, a few barley lines with naked caryopses were created using the genetic background of the hulled cultivar Golden Promise. These lines can further be utilized as experimental material for reverse genetics studies on the molecular function of the *NUD* gene and its possible pleiotropic effects. The newly created *NUD* knockout lines may be of unprecedented utility for such investigations than previously available, chemically or X-ray-induced mutants or near-isogenic lines. The *NUD* gene-specific gRNAs developed in the present study were demonstrated to be exceedingly efficient in inducing KO mutations in highly conserved target motifs and thus can be readily used for any transformable barley cultivar in order to convert it from hulled to naked grain type. This approach may be beneficial for the development of functional food in various global regions including those where naked barley has not been traditionally used as food.

## Methods

### Guide RNA design and vector construction

Target motif selection was performed using the online tools DESKGEN [41] and WU-CRISPR [42]. Guide-RNA secondary structures were modelled using the RNAfold tool (http://rna.tbi.univie.ac.at/cgi-bin/RNAWebSuite/RNAfold.cgi [38]). Off-target analysis was performed using the DESKGEN tool and via BLAST of target sequences at the IPK Barley BLAST Server (https://webblast.ipk-gatersleben.de/barley_ibsc/).

The nucleotide sequence of the *NUD* gene target fragment in the genome of barley cv. Golden Promise was confirmed by PCR with the primers Hv_Nud_F and Hv_Nud_R2 (Additional file 5, Table S5) and Sanger sequencing. Four vectors harboring *cas9* and gRNA expression units were constructed on the basis of the pSH121 generic vector (Additional file 6, Figure S1) so as to address four target motifs within the *HvNUD* coding sequence (Fig. 1a). The vector construction was performed as described previously [15] using pairs of oligonucleotides listed in (Additional file 5: Table S5).

### Prevalidation of gRNA activity by ballistic DNA transfer to leaf epidermis cells

To reveal the best performing gRNAs, a transient expression-based test for cleavage activity of Cas9/gRNA was performed as previously described [16] for each created *cas9*/gRNA construct. TARGET vectors were created using the generic vector pNB1 (GenBank: KU705395) as a backbone. The double-stranded oligonucleotides (Additional file 5, Table S5) corresponding to each target site with PAM were inserted in the pNB1 vector between the *Bam*HI and *Eco*RI sites. In total, four TARGET vectors were created.

Leaf explants were taken from 10 days-old barley plants (cv. Golden Promise) grown in a glasshouse. Plasmid DNA was mixed using a total volume of 10 μl in the following proportions: 7 μg of target vector, 7 μg of *cas9*/gRNA vector, 2 μg of mCherry vector [16]. As positive control, a mixture of a pNB1 vector variant carrying an intact *YFP* expression cassette and mCherry vector was used, whereas a mixture of one of the target vectors and mCherry vector served as negative control. The coating of gold particles with plasmid DNA and bombardment were performed as previously described [16]. For each combination of constructs, two independent experiments were conducted, with two technical replicates per experiment (i.e. four in total). After bombardment, explants were kept in the dark for 28–48 h. Reporter gene expression was observed with a Zeiss CLSM780 confocal laser scanning microscope using a 561 nm Helium-Neon-laser for mCherry detection (emission at 610 nm) and a 488 nm Argon-laser for YFP detection (emission at 527 nm). Activity of each *cas9*/gRNA construct was deduced from the ratio between the number of cells accumulating both YFP and mCherry (YFP cells) and the number of transgenic cells (mCherry cells).

### Prevalidation of gRNA activity by protoplast transfection

Leaf mesophyll protoplasts were isolated from etiolated barley seedlings (cv. Golden Promise) and co-transfected with one of the selected *cas9*/gRNA vectors and the *GFP*-expressing control vector pYF133 [43]. Transformed protoplasts were incubated in the dark at 21 °C for 2 days. After determination of the portion of GFP-positive cells, genomic DNA was isolated from protoplasts and the target region was amplified using primers Hv_Nud_F4 - Hv_Nud_1exR (Additional file 5, Table S5). The mutation frequency and pattern in protoplasts were examined by deep amplicon sequencing. The proportion of GFP-positive cells was used to normalize the mutagenic activity of the respective *cas9*/gRNA vector. The non-parametric Kolmogorov-Smirnov test was used to compare the distributions of mutation types induced by selected gRNAs. Protoplast isolation, transformation, and amplicon sequencing were performed as described previously [15].

### Plant material

The experiments of this investigation were conducted using the two-rowed spring-type barley (*Hordeum vulgare* L.) British cv. “Golden Promise” which is an X-ray mutant derived from its predecessor cv. “Maythorpe”. The barley plants were cultivated as described previously [44].

### Binary vector construction and Agrobacterium-mediated barley transformation

The *cas9* and selected gRNA expression units were transferred as *Sfi*I fragments from the aforementioned pSH121-derived plasmids to the generic binary vector p6i-2x35s-TE9 (DNA Cloning Service, Hamburg, Germany), which resulted in the generation of the NUD-45 and NUD-50 transformation vectors. These vectors were transferred into *A. tumefaciens* strain AGL1 by electroporation. *Agrobacterium*-mediated gene transfer to immature embryos (barley cv. Golden Promise) was performed following a method described previously [44, 45]. The primary transgenic plants were selected using 50 mg/l hygromycin and were then investigated by PCR for the presence of *cas9* (Bie475 and zCas9-R1), gRNA (OsU3p-F1 and guide-specific RGEN Rev. Oligo) and progeny were additionally tested for the *hygromycin phosphotransferase* gene used as selectable marker (35S-F2 and HYG-R5). Primer structures are given in (Additional file 5, Table S5).

### Mutation detection in primary transgenic plants

Mutations were detected by Sanger sequence data analysis. To this end, genomic DNA was extracted from candidate plants [46] and target and off-target regions were amplified and sequenced using the primers listed in Additional file 1, Table S5. Sanger chromatograms were analyzed for the presence of nucleotide sequence changes or abnormalities in the target motif.

### Genotyping and phenotyping of progeny

Progeny of primary plants were growing for 1 month under standard glasshouse conditions (16 h, 18 °C/ 8 h, 16 °C) and later transferred to speed breeding conditions (22 h, 21 °C/ 2 h, 17 °C) for 2.5 months. DNA was isolated from 1 week-old seedlings by using 96-well format DNA isolation [47]. Genotyping of target and off-target regions was done by PCR amplification and Sanger sequencing. After 3.5 months, fully matured spikes were harvested. As a standardized procedure, a hand thresher (Dreschhexe, Baumann Saatzuchbedarf, Germany) was used to remove the hulls from the spikes. For this, five spikes per line were threshed four times and grains were collected. Remaining hulls were removed by hand (Fig. 5b).

## Supplementary information

**Additional file 1: Supplementary Table S1.** Guide RNA structure and activity in the transient expression test.

**Additional file 2: Supplementary Table S2.** Evaluation of protoplast transfection efficiency.

**Additional file 3: Supplementary Table S3.** Deep-sequencing results for target motifs in mutated protoplast population.

**Additional file 4: Supplementary Table S4.** Target mutations in T0 and T1 detected in selected plants.

**Additional file 5: Supplementary Table S5.** List of primers and oligonucleotides used in this study.

**Additional file 6: Supplementary Figure S1.** Sequence data and map of generic vector pSH121

## Data Availability

All data generated or analyzed during this study are included in this published article and its supplementary materials file or are freely accessible in a data repository (plasmid pNB1: GenBank accession KU705395). Materials generated in this study are available from the corresponding authors upon reasonable request.

## Abbreviations

ERF: Ethylene Response Factor
KO: Knockout
LDL-cholesterol: Low-density lipoprotein cholesterol
PAM: Protospacer-adjacent motif
QTL: Quantitative trait locus
WIN1/SHN1: Wax Inducer 1/Shine 1
