# Supplementary material for: Conversion of hulled into naked barley by Cas endonuclease-mediated knockout of the NUD gene
Source: BMC Plant Biol. 2020 Oct 14;20(Suppl 1):255. doi: 10.1186/s12870-020-02454-9 (PMC7556925; doi:10.1186/s12870-020-02454-9)
Supplement: Supplementary file 1 — Additional file 1: Supplementary Table S1. Guide RNA structure and activity in the transient expression test. [file 12870_2020_2454_MOESM1_ESM.docx]

**Supplementary Table S1.** Guide RNA structure and activity in transient expression test

| **gRNA** | **Structure of target-specific region of gRNA (5’-3’)** | **mCherry cells** | **YFP cells** | **Ratio of YFP/mCherry cells** |
| --- | --- | --- | --- | --- |
| Nud14 | AGAAGAAGTTTCGCGGCGTC | 152 | 48 | 0.32 |
| Nud45 | GGAGACCCAGGAGCCCCAG | 164 | 87 | 0.53 |
| Nud50 | GCTCCTGGGTCTCCGAGATC | 112 | 30 | 0.27 |
| Nud180 | AGACCAACTTCCCCGTACCG | 195 | 71 | 0.36 |
