# Supplementary material for: Conversion of hulled into naked barley by Cas endonuclease-mediated knockout of the NUD gene
Source: BMC Plant Biol. 2020 Oct 14;20(Suppl 1):255. doi: 10.1186/s12870-020-02454-9 (PMC7556925; doi:10.1186/s12870-020-02454-9)
Supplement: Supplementary file 2 — Additional file 2: Supplementary Table S2. Evaluation of protoplast transfection efficiency. [file 12870_2020_2454_MOESM2_ESM.docx]

**Supplementary Table S2.** Evaluation of protoplast transformation efficiency

| **gRNA** | **cells total** | **GFP positive cells** | **transformation efficiency** |
| --- | --- | --- | --- |
| Nud45 | 451 | 216 | 0.48 |
| Nud50 | 32 | 23 | 0.72 |
