# Supplementary material for: Conversion of hulled into naked barley by Cas endonuclease-mediated knockout of the NUD gene
Source: BMC Plant Biol. 2020 Oct 14;20(Suppl 1):255. doi: 10.1186/s12870-020-02454-9 (PMC7556925; doi:10.1186/s12870-020-02454-9)
Supplement: Supplementary file 3 — Additional file 3: Supplementary Table S3. Deep-sequencing results for target motifs in mutated protoplast population. [file 12870_2020_2454_MOESM3_ESM.docx]

**Supplementary Table S3.** Deep-sequencing results for target motifs in mutated protoplast population.

Nud45 and Nud50 target motifs

| **Mutation type [bp]** | **Number of reads** | |
| --- | --- | --- |
|  | **Nud45** | **Nud50** |
| +1 | 424 | 0 |
| WT | 4,185 | 1,823 |
| -1 | 2,441 | 527 |
| -2 | 967 | 0 |
| -3 | 231 | 0 |
| -4 | 179 | 615 |
| -5 | 0 | 102 |
| -6 | 118 | 141 |
| -7 | 107 | 549 |
| -8 | 227 | 0 |
| -9 | 211 | 0 |
| total | 9,090 | 3,757 |
