# Supplementary material for: Conversion of hulled into naked barley by Cas endonuclease-mediated knockout of the NUD gene
Source: BMC Plant Biol. 2020 Oct 14;20(Suppl 1):255. doi: 10.1186/s12870-020-02454-9 (PMC7556925; doi:10.1186/s12870-020-02454-9)
Supplement: Supplementary file 4 — Additional file 4: Supplementary Table S4. Target mutations in T0 and T1 detected in selected plants. [file 12870_2020_2454_MOESM4_ESM.docx]

**Supplementary Table S4.** Target mutations in T0 and T1 detected in selected plants

| **Primary transgenic plants (T0)** | ***NUD* alleles detected**  **in T0*** | **T0 phenotype** | ***NUD* alleles detected in T1** | **No. of**  **T1 plants** |
| --- | --- | --- | --- | --- |
| Nud45 plant 04 | -214+5 bp | naked | -214+5 bp homozygous | 4 |
| Nud45 plant 14 | -3 bp | naked/hulled | -3 bp homozygous | 2 |
| Nud45 plant 27 | -1 bp | naked/hulled | -1 bp homozygous | 5 |
| Nud50 plant 16 | -3 bp  -17 bp | naked | -3 bp homozygous  -17 bp homozygous  Heterozygous | 6  8  11 |
| Nud50 plant 31 | +1 bp  -6 bp | naked/hulled | WT homozygous  +1 bp homozygous  -6 bp homozygous | 1  5  1 |
| Nud50 plant 33 | WT  -4 bp | naked/hulled | -1 bp homozygous  -19 bp homozygous  Heterozygous | 6  1  15 |

*) since primary mutant plants can be chimeric (including various sectors with homozygous, heterozygous and biallelic mutations as well as WT alleles) and the leaf samples analysed are not necessarily representative for the entire plant, it is impossible to deduce conclusions on zygosity of the detected mutations in this generation
