# Supplementary material for: Conversion of hulled into naked barley by Cas endonuclease-mediated knockout of the NUD gene
Source: BMC Plant Biol. 2020 Oct 14;20(Suppl 1):255. doi: 10.1186/s12870-020-02454-9 (PMC7556925; doi:10.1186/s12870-020-02454-9)
Supplement: Supplementary file 5 — Additional file 5: Supplementary Table S5. List of primers and oligonucleotides used in this study. [file 12870_2020_2454_MOESM5_ESM.docx]

**Supplementary Table S5.** List of primers and oligonucleotides

| **Indicator** | **DNA sequence (5’-3’)** | **Locus** |
| --- | --- | --- |
| Hv_Nud_F1 | TGACGCACATGACAGATTGAC | HORVU7Hr1G089930 (Nud) |
| Hv_Nud_R2 | CAATGTGGGACTTCTCGGTG | HORVU7Hr1G089930 (Nud) |
| Hv_Nud_1exR | GAGTCGTAGGCCGGAGAGTAG | HORVU7Hr1G089930 (Nud) |
| Hv_Nud_F4 | ACTGCCTGCTGATCATTCCT | HORVU7Hr1G089930 (Nud) |
| Win1_F | CGTACTAAGTGACTCCAGCAG | HORVU6Hr1G038120 (off-target) |
| Win1_R | TAAACCGGCGAAGAAACCAC | HORVU6Hr1G038120 (off-target) |
| 7H_gene_F | TTTGCCAACTAAACGTGTCACC | HORVU7Hr1G029870 (off-target) |
| 7H_gene_R | CAATCGCAGAGCCTTTCGAC | HORVU7Hr1G029870 (off-target) |
| 6H_gene_F | GAGAAGTCCGAGTCTAGCAG | HORVU6Hr1G085850 (off-target) |
| 6H_gene_R | AAACGGGCTAAACAAAGATGTC | HORVU6Hr1G085850 (off-target) |
| Bie475 | TTTAGCCCTGCCTTCATACG | ZmUbi1-promotor (cas9) |
| zCas9-R1 | TTAATCATGTGGGCCAGAGC | Spcas9 (maize codon-optimized) |
| OsU3p-F1 | CAGGGACCATAGCACAAGAC | OsU3-promotor (gRNA) |
| 35S-F2 | CATGGTGGAGCACGACACTCTC | 35S-promotor (Hygromycin) |
| HYG-R5 | GATTCCTTGCGGTCCGAATG | Hygromycin phosphotransferase |

| **cas9/gRNA vector** | **gRNA Fwd oligo 5’-3’** | **gRNA Rev oligo 5’-3’** |
| --- | --- | --- |
| RGEN-Nud14 | TGGCAGAAGAAGTTTCGCGGCGTC | AAACGACGCCGCGAAACTTCTTCT |
| RGEN-Nud45 | TGGCGGAGACCCAGGAGCCCCAG | AAACCTGGGGCTCCTGGGTCTCC |
| RGEN-Nud50 | TGGCGCTCCTGGGTCTCCGAGATC | AAACGATCTCGGAGACCCAGGAGC |
| RGN-Nud180 | TGGCAGACCAACTTCCCCGTACCG | AAACCGGTACGGGGAAGTTGGTCT |

| **TARGET**  **-vector** | **Target Fwd oligo 5’-3’** | **Target Rev oligo 5’-3’** |
| --- | --- | --- |
| T-Nud14 | GATCAGAAGAAGTTTCGCGGCGTCAGGC | AATTGCCTGACGCCGCGAAACTTCTTCT |
| T-Nud45 | GATCCGGAGACCCAGGAGCCCCAGTGGC | AATTGCCACTGGGGCTCCTGGGTCTCCG |
| T-Nud50 | GATCGCTCCTGGGTCTCCGAGATCAGGC | AATTGCCTGATCTCGGAGACCCAGGAGC |
| T-Nud180 | GATCAGACCAACTTCCCCGTACCGAGGC | AATTGCCTCGGTACGGGGAAGTTGGTCT |
