# Supplementary material for: Conversion of hulled into naked barley by Cas endonuclease-mediated knockout of the NUD gene
Source: BMC Plant Biol. 2020 Oct 14;20(Suppl 1):255. doi: 10.1186/s12870-020-02454-9 (PMC7556925; doi:10.1186/s12870-020-02454-9)
Supplement: Supplementary file 6 — Additional file 6: Supplementary Figure S1. Sequence data and map of generic vector pSH121 [file 12870_2020_2454_MOESM6_ESM.docx]

**Supplementary Figure S1.** Sequence data and map of generic vector pSH121


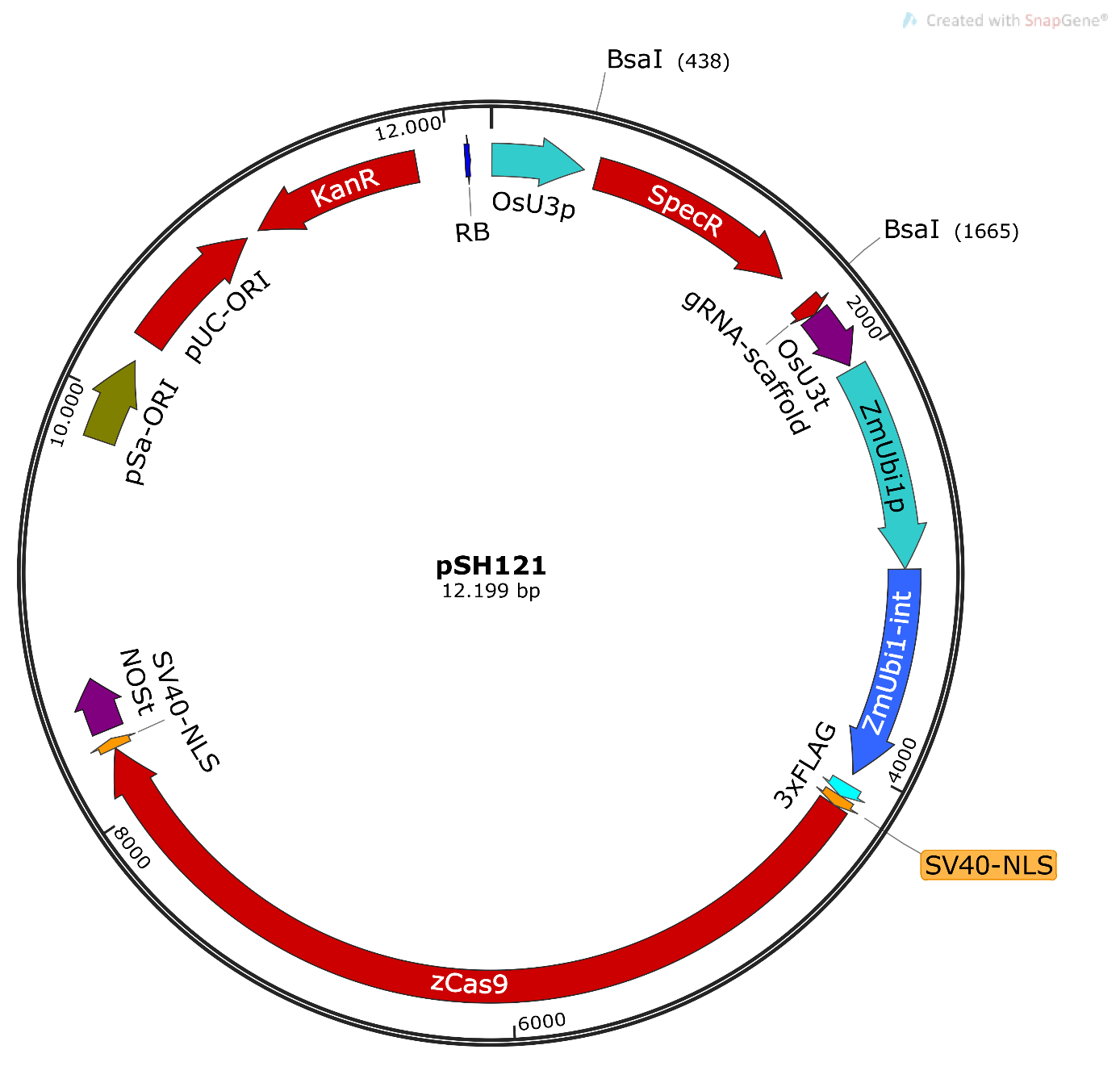


| **Feature** | **Description** | **Start – End** |
| --- | --- | --- |
| OsU3p | Rice U3 promotor | 6 – 442 bp |
| SpecR | Spectinomycin resistance gene | 504 – 1514 bp |
| gRNA-scaffold | gRNA-scaffold sequence | 1666 – 1741 bp |
| OsU3t | Rice U3 terminator | 1742 – 2032 bp |
| ZmUbi1p | Maize POLYUBIQUITIN 1 promotor | 2051 – 3031 bp |
| ZmUbi1-int | First Intron of Maize UBIQUITIN 1 | 3032 – 4035 bp |
| 3xFLAG | 3x FLAG-tag | 4087 – 4152 bp |
| SV40-NLS | NLS derived from SV40 T antigen | 4153 – 4203 bp |
| zCas9 | Maize codon-optimized SpCas9 | 4204 – 8304 bp |
| SV40-NLS | NLS derived from SV40 T antigen | 8305 – 8352 bp |
| NosT | Nopaline synthase terminator | 8397 – 8650 bp |
| pSa-ORI | pSa origin of replication | 9785 – 10195 bp |
| pUC-ORI | pUC origin of replication | 10305 – 10979 bp |
| KanR | Kanamycin resistance gene | 11851 – 11042 bp |
| RB | Right border | 12080 – 12103 bp |

1 agcttagtaa ttcatccagg tcaccaagtt ctaggatttt cagaactgca acttatttta tcaaggaatc

71 tttaaacata cgaacagatc acttaaagtt cttctgaagc aacttaaagt tatcaggcat gcatggatct

141 tggaggaatc agatgtgcag tcagggacca tagcacaaga caggcgtctt ctactggtgc taccagcaaa

211 tgctggaagc cgggaacact gggtacgttg gaaaccacgt gatgtgaaga agtaagataa actgtaggag

281 aaaagcattt cgtagtgggc catgaagcct ttcaggacat gtattgcagt atgggccggc ccattacgca

351 attggacgac aacaaagact agtattagta ccacctcggc tatccacata gatcaaagct gatttaaaag

421 agttgtgcag atgatccgtg gcggagaccc caacccagtg gacataagcc tgttcggttc gtaagctgta

491 atgcaagtag cgtatgcgct cacgcaactg gtccagaacc ttgaccgaac gcagcggtgg taacggcgca

561 gtggcggttt tcatggcttg ttatgactgt ttttttgggg tacagtctat gcctcgggca tccaagcagc

631 aagcgcgtta cgccgtgggt cgatgtttga tgttatggag cagcaacgat gttacgcagc agggcagtcg

701 ccctaaaaca aagttaaaca tcatggggga agcggtgatc gccgaagtat cgactcaact atcagaggta

771 gttggcgtca tcgagcgcca tctcgaaccg acgttgctgg ccgtacattt gtacggctcc gcagtggatg

841 gcggcctgaa gccacacagt gatattgatt tgctggttac ggtgaccgta aggcttgatg aaacaacgcg

911 gcgagctttg atcaacgacc ttttggaaac ttcggcttcc cctggagaga gcgagattct ccgcgctgta

981 gaagtcacca ttgttgtgca cgacgacatc attccgtggc gttatccagc taagcgcgaa ctgcaatttg

1051 gagaatggca gcgcaatgac attcttgcag gtatcttcga gccagccacg atcgacattg atctggctat

1121 cttgctgaca aaagcaagag aacatagcgt tgccttggta ggtccagcgg cggaggaact ctttgatccg

1191 gttcctgaac aggatctatt tgaggcgcta aatgaaacct taacgctatg gaactcgccg cccgactggg

1261 ctggcgatga gcgaaatgta gtgcttacgt tgtcccgcat ttggtacagc gcagtaaccg gcaaaatcgc

1331 gccgaaggat gtcgctgccg actgggcaat ggagcgcctg ccggcccagt atcagcccgt catacttgaa

1401 gctagacagg cttatcttgg acaagaagaa gatcgcttgg cctcgcgcgc agatcagttg gaagaatttg

1471 tccactacgt gaaaggcgag atcaccaagg tagtcggcaa ataatgtcta gctagaaatt cgttcaagcc

1541 gacgccgctt cgcggcgcgg cttaactcaa gcgttagatg cactaagcac ataattgctc acagccaaac

1611 tatcaggtca agtctgcttt tattattttt aagcgtgcat aataagccgg tctcggtttt agagctagaa

1681 atagcaagtt aaaataaggc tagtccgtta tcaacttgaa aaagtggcac cgagtcggtg cttttttttt

1751 tcgttttgca ttgagttttc tccgtcgcat gtttgcagtt ttattttccg ttttgcattg aaatttctcc

1821 gtctcatgtt tgcagcgtgt tcaaaaagta cgcagctgta tttcacttat ttacggcgcc acattttcat

1891 gccgtttgtg ccaactatcc cgagctagtg aatacagctt ggcttcacac aacactggtg acccgctgac

1961 ctgctcgtac ctcgtaccgt cgtacggcac agcatttgga attaaagggt gtgatcgata ctgcttgctg

2031 ctaagcttga tatcgaattc ctgcagtgca gcgtgacccg gtcgtgcccc tctctagaga taatgagcat

2101 tgcatgtcta agttataaaa aattaccaca tatttttttt gtcacacttg tttgaagtgc agtttatcta

2171 tctttataca tatatttaaa ctttactcta cgaataatat aatctatagt actacaataa tatcagtgtt

2241 ttagagaatc atataaatga acagttagac atggtctaaa ggacaattga gtattttgac aacaggactc

2311 tacagtttta tctttttagt gtgcatgtgt tctccttttt ttttgcaaat agcttcacct atataatact

2381 tcatccattt tattagtaca tccatttagg gtttagggtt aatggttttt atagactaat ttttttagta

2451 catctatttt attctatttt agcctctaaa ttaagaaaac taaaactcta ttttagtttt tttatttaat

2521 aatttagata taaaatagaa taaaataaag tgactaaaaa ttaaacaaat accctttaag aaattaaaaa

2591 aactaaggaa acatttttct tgtttcgagt agataatgcc agcctgttaa acgccgtcga cgagtctaac

2661 ggacaccaac cagcgaacca gcagcgtcgc gtcgggccaa gcgaagcaga cggcacggca tctctgtcgc

2731 tgcctctgga cccctctcga gagttccgct ccaccgttgg acttgctccg ctgtcggcat ccagaaatgc

2801 gtggcggagc ggcagacgtg agccggcacg gcaggcggcc tcctcctcct ctcacggcac ggcagctacg

2871 ggggattcct ttcccaccgc tccttcgctt tcccttcctc gcccgccgta ataaatagac accccctcca

2941 caccctcttt ccccaacctc gtgttgttcg gagcgcacac acacacaacc agatctcccc caaatccacc

3011 cgtcggcacc tccgcttcaa ggtacgccgc tcgtcctccc cccccccccc tctctacctt ctctagatcg

3081 gcgttccggt ccatggttag ggcccggtag ttctacttct gttcatgttt gtgttagatc cgtgtttgtg

3151 ttagatccgt gctgctagcg ttcgtacacg gatgcgacct gtacgtcaga cacgttctga ttgctaactt

3221 gccagtgttt ctctttgggg aatcctggga tggctctagc cgttccgcag acgggatcga tttcatgatt

3291 ttttttgttt cgttgcatag ggtttggttt gcccttttcc tttatttcaa tatatgccgt gcacttgttt

3361 gtcgggtcat cttttcatgc ttttttttgt cttggttgtg atgatgtggt ctggttgggc ggtcgttcta

3431 gatcggagta gaattctgtt tcaaactacc tggtggattt attaattttg gatctgtatg tgtgtgccat

3501 acatattcat agttacgaat tgaagatgat ggatggaaat atcgatctag gataggtata catgttgatg

3571 cgggttttac tgatgcatat acagagatgc tttttgttcg cttggttgtg atgatgtggt gtggttgggc

3641 ggtcgttcat tcgttctaga tcggagtaga atactgtttc aaactacctg gtgtatttat taattttgga

3711 actgtatgtg tgtgtcatac atcttcatag ttacgagttt aagatggatg gaaatatcga tctaggatag

3781 gtatacatgt tgatgtgggt tttactgatg catatacatg atggcatatg cagcatctat tcatatgctc

3851 taaccttgag tacctatcta ttataataaa caagtatgtt ttataattat tttgatcttg atatacttgg

3921 atgatggcat atgcagcagc tatatgtgga tttttttagc cctgccttca tacgctattt atttgcttgg

3991 tactgtttct tttgtcgatg ctcaccctgt tgtttggtgt tacttctgca gcccggggga tccccaatac

4061 ttgtatggcc gcggccgctc tagatggatt acaaggacca cgacggggat tacaaggacc acgacattga

4131 ttacaaggat gatgatgaca agatggctcc gaagaagaag aggaaggttg gcatccacgg ggtgccagct

4201 gctgacaaga agtactcgat cggcctcgat attgggacta actctgttgg ctgggccgtg atcaccgacg

4271 agtacaaggt gccctcaaag aagttcaagg tcctgggcaa caccgatcgg cattccatca agaagaatct

4341 cattggcgct ctcctgttcg acagcggcga gacggctgag gctacgcggc tcaagcgcac cgcccgcagg

4411 cggtacacgc gcaggaagaa tcgcatctgc tacctgcagg agattttctc caacgagatg gcgaaggttg

4481 acgattcttt cttccacagg ctggaggagt cattcctcgt ggaggaggat aagaagcacg agcggcatcc

4551 aatcttcggc aacattgtcg acgaggttgc ctaccacgag aagtacccta cgatctacca tctgcggaag

4621 aagctcgtgg actccacaga taaggcggac ctccgcctga tctacctcgc tctggcccac atgattaagt

4691 tcaggggcca tttcctgatc gagggggatc tcaacccgga caatagcgat gttgacaagc tgttcatcca

4761 gctcgtgcag acgtacaacc agctcttcga ggagaacccc attaatgcgt caggcgtcga cgcgaaggct

4831 atcctgtccg ctaggctctc gaagtctcgg cgcctcgaga acctgatcgc ccagctgccg ggcgagaaga

4901 agaacggcct gttcgggaat ctcattgcgc tcagcctggg gctcacgccc aacttcaagt cgaatttcga

4971 tctcgctgag gacgccaagc tgcagctctc caaggacaca tacgacgatg acctggataa cctcctggcc

5041 cagatcggcg atcagtacgc ggacctgttc ctcgctgcca agaatctgtc ggacgccatc ctcctgtctg

5111 atattctcag ggtgaacacc gagattacga aggctccgct ctcagcctcc atgatcaagc gctacgacga

5181 gcaccatcag gatctgaccc tcctgaaggc gctggtcagg cagcagctcc ccgagaagta caaggagatc

5251 ttcttcgatc agtcgaagaa cggctacgct gggtacattg acggcggggc ctctcaggag gagttctaca

5321 agttcatcaa gccgattctg gagaagatgg acggcacgga ggagctgctg gtgaagctca atcgcgagga

5391 cctcctgagg aagcagcgga cattcgataa cggcagcatc ccacaccaga ttcatctcgg ggagctgcac

5461 gctatcctga ggaggcagga ggacttctac cctttcctca aggataaccg cgagaagatc gagaagattc

5531 tgactttcag gatcccgtac tacgtcggcc cactcgctag gggcaactcc cgcttcgctt ggatgacccg

5601 caagtcagag gagacgatca cgccgtggaa cttcgaggag gtggtcgaca agggcgctag cgctcagtcg

5671 ttcatcgaga ggatgacgaa tttcgacaag aacctgccaa atgagaaggt gctccctaag cactcgctcc

5741 tgtacgagta cttcacagtc tacaacgagc tgactaaggt gaagtatgtg accgagggca tgaggaagcc

5811 ggctttcctg tctggggagc agaagaaggc catcgtggac ctcctgttca agaccaaccg gaaggtcacg

5881 gttaagcagc tcaaggagga ctacttcaag aagattgagt gcttcgattc ggtcgagatc tctggcgttg

5951 aggaccgctt caacgcctcc ctggggacct accacgatct cctgaagatc attaaggata aggacttcct

6021 ggacaacgag gagaatgagg atatcctcga ggacattgtg ctgacactca ctctgttcga ggaccgggag

6091 atgatcgagg agcgcctgaa gacttacgcc catctcttcg atgacaaggt catgaagcag ctcaagagga

6161 ggaggtacac cggctggggg aggctgagca ggaagctcat caacggcatt cgggacaagc agtccgggaa

6231 gacgatcctc gacttcctga agagcgatgg cttcgcgaac cgcaatttca tgcagctgat tcacgatgac

6301 agcctcacat tcaaggagga tatccagaag gctcaggtga gcggccaggg ggactcgctg cacgagcata

6371 tcgcgaacct cgctggctcg ccagctatca agaaggggat tctgcagacc gtgaaggttg tggacgagct

6441 ggtgaaggtc atgggcaggc acaagcctga gaacatcgtc attgagatgg cccgggagaa tcagaccacg

6511 cagaagggcc agaagaactc acgcgagagg atgaagagga tcgaggaggg cattaaggag ctggggtccc

6581 agatcctcaa ggagcacccg gtggagaaca cgcagctgca gaatgagaag ctctacctgt actacctcca

6651 gaatggccgc gatatgtatg tggaccagga gctggatatt aacaggctca gcgattacga cgtcgatcat

6721 atcgttccac agtcattcct gaaggatgac tccattgaca acaaggtcct caccaggtcg gacaagaacc

6791 ggggcaagtc tgataatgtt ccttcagagg aggtcgttaa gaagatgaag aactactggc gccagctcct

6861 gaatgccaag ctgatcacgc agcggaagtt cgataacctc acaaaggctg agaggggcgg gctctctgag

6931 ctggacaagg cgggcttcat caagaggcag ctggtcgaga cacggcagat cactaagcac gttgcgcaga

7001 ttctcgactc acggatgaac actaagtacg atgagaatga caagctgatc cgcgaggtga aggtcatcac

7071 cctgaagtca aagctcgtct ccgacttcag gaaggatttc cagttctaca aggttcggga gatcaacaat

7141 taccaccatg cccatgacgc gtacctgaac gcggtggtcg gcacagctct gatcaagaag tacccaaagc

7211 tcgagagcga gttcgtgtac ggggactaca aggtttacga tgtgaggaag atgatcgcca agtcggagca

7281 ggagattggc aaggctaccg ccaagtactt cttctactct aacattatga atttcttcaa gacagagatc

7351 actctggcca atggcgagat ccggaagcgc cccctcatcg agacgaacgg cgagacgggg gagatcgtgt

7421 gggacaaggg cagggatttc gcgaccgtca ggaaggttct ctccatgcca caagtgaata tcgtcaagaa

7491 gacagaggtc cagactggcg ggttctctaa ggagtcaatt ctgcctaagc ggaacagcga caagctcatc

7561 gcccgcaaga aggactggga tccgaagaag tacggcgggt tcgacagccc cactgtggcc tactcggtcc

7631 tggttgtggc gaaggttgag aagggcaagt ccaagaagct caagagcgtg aaggagctgc tggggatcac

7701 gattatggag cgctccagct tcgagaagaa cccgatcgat ttcctggagg cgaagggcta caaggaggtg

7771 aagaaggacc tgatcattaa gctccccaag tactcactct tcgagctgga gaacggcagg aagcggatgc

7841 tggcttccgc tggcgagctg cagaagggga acgagctggc tctgccgtcc aagtatgtga acttcctcta

7911 cctggcctcc cactacgaga agctcaaggg cagccccgag gacaacgagc agaagcagct gttcgtcgag

7981 cagcacaagc attacctcga cgagatcatt gagcagattt ccgagttctc caagcgcgtg atcctggccg

8051 acgcgaatct ggataaggtc ctctccgcgt acaacaagca ccgcgacaag ccaatcaggg agcaggctga

8121 gaatatcatt catctcttca ccctgacgaa cctcggcgcc cctgctgctt tcaagtactt cgacacaact

8191 atcgatcgca agaggtacac aagcactaag gaggtcctgg acgcgaccct catccaccag tcgattaccg

8261 gcctctacga gacgcgcatc gacctgtctc agctcggggg cgacaagcgg ccagcggcga cgaagaaggc

8331 ggggcaggcg aagaagaaga agtgagctca ggcctccata caagtattgg ggatccgaat ttccccgatc

8401 gttcaaacat ttggcaataa agtttcttaa gattgaatcc tgttgccggt cttgcgatga ttatcatata

8471 atttctgttg aattacgtta agcatgtaat aattaacatg taatgcatga cgttatttat gagatgggtt

8541 tttatgatta gagtcccgca attatacatt taatacgcga tagaaaacaa aatatagcgc gcaaactagg

8611 ataaattatc gcgcgcggtg tcatctatgt tactagatcg ggaattcact ggccgtcgtt ttacaacgtc

8681 gtgactggga aaaccctggc gttacccaac ttaatcgcct tgcagcacat ccccctttcg ccaggggtac

8751 caggccgcca tggccaggta ccgccccgtc cggtcctgcc cgtcaccgag atttgactcg agtttctcca

8821 taataatgtg tgagtagttc ccagataagg gaattagggt tcctataggg tttcgctcat gtgttgagca

8891 tataagaaac ccttagtatg tatttgtatt tgtaaaatac ttctatcaat aaaatttcta attcctaaaa

8961 ccaaaatcca gtactaaaat ccagatcccc cgaattaatt cggcgttaat tcagtacatt aaaaacgtcc

9031 gcaatgtgtt attaagttgt cactagtcag gttaactcaa ttcggcgtta attcagtaca ttaaaaacgt

9101 ccgcaatgtg ttattaagtt gtctaagcgt caatttgttt acaccacaat atatcctgcc accagccagc

9171 caacagctcc ccgaccggca gctcggcaca aaatcaccac tcgatacagg cagcccatca gtccgggacg

9241 gcgtcagcgg gagagccgtt gtaaggcggc agactttgct catgttaccg atgctattcg gaagaacggc

9311 aactaagctg ccgggtttga aacacggatg atctcgcgga gggtagcatg ttgattgtaa cgatgacaga

9381 gcgttgctgc ctgtgatcac ttaagtaact aactaacagg aagagtttgt agaaacgcaa aaaggccatc

9451 cgtcaggatg gccttctgct tagtttgatg cctggcagtt tatggcgggc gtcctgcccg ccaccctccg

9521 ggccgttgct tcacaacgtt caaatccgct cccggcggat ttgtcctact caggagagcg ttcaccgaca

9591 aacaacagat aaaacgaaag gcccagtctt ccgactgagc ctttcgtttt atttgatgcc tggcagttcc

9661 ctactctcgc ttagtagtta gacgtccccg agatccatgc tagaccatga atccagaagc ccgagaggtt

9731 gccgcctttc gggctttttc tttttcaaaa aaaaaaattt ataaaacgat ctgttgcggc cggccgccgg

9801 gttgtgggca aaggcgctcg acggtgggca accgcttgcg gttgtccacg ggcggagccg gtgcgcgtag

9871 cgcattgtcc acaagccaag ggcgaccaat aattgatata tatattcata attgaaaagc taattgaaca

9941 tactacttgc tgtaactact tgccggagcg aggggtgttt gcaagctgtt gatctgaaag ggctattagc

10011 gttctcacgt gcctttttga ttagcgattt cacgtgacct tattagcgat ttcacgtact ccgattagcg

10081 atttcacgta ccctgattag cgatttcacg tggatagttt ttggagcggg ccggaaagcc ccgtgaatca

10151 aggctttgcg gggcattagc ggtttcacgt ggataactac cctctatcca caggcttccg gggataaaaa

10221 agcccgctcg acggcgggct gttggatggg gatctagcgg taatacggtt atccacagaa tcaggggata

10291 acgcaggaaa gaacatgtga gcaaaaggcc agcaaaaggc caggaaccgt aaaaaggccg cgttgctggc

10361 gtttttccat aggctccgcc cccctgacga gcatcacaaa aatcgacgct caagtcagag gtggcgaaac

10431 ccgacaggac tataaagata ccaggcgttt ccccctggaa gctccctcgt gcgctctcct gttccgaccc

10501 tgccgcttac cggatacctg tccgcctttc tcccttcggg aagcgtggcg ctttctcata gctcacgctg

10571 taggtatctc agttcggtgt aggtcgttcg ctccaagctg ggctgtgtgc acgaaccccc cgttcagccc

10641 gaccgctgcg ccttatccgg taactatcgt cttgagtcca acccggtaag acacgactta tcgccactgg

10711 cagcagccac tggtaacagg attagcagag cgaggtatgt aggcggtgct acagagttct tgaagtggtg

10781 gcctaactac ggctacacta gaagaacagt atttggtatc tgcgctctgc tgaagccagt taccttcgga

10851 aaaagagttg gtagctcttg atccggcaaa caaaccaccg ctggtagcgg tggttttttt gtttgcaagc

10921 agcagattac gcgcagaaaa aaaggatctc aagaagatcc tttgatcttt tctaccgggt ctgacgctca

10991 gtggaacggg gcccaatctg aataatgtta caaccaatta accaattctg attagaaaaa ctcatcgagc

11061 atcaaatgaa actgcaattt attcatatca ggattatcaa taccatattt ttgaaaaagc cgtttctgta

11131 atgaaggaga aaactcaccg aggcagttcc ataggatggc aagatcctgg tatcggtctg cgattccgac

11201 tcgtccaaca tcaatacaac ctattaattt cccctcgtca aaaataaggt tatcaagtga gaaatcacca

11271 tgagtgacga ctgaatccgg tgagaatggc aaaagtttat gcatttcttt ccagacttgt tcaacaggcc

11341 agccattacg ctcgtcatca aaatcactcg catcaaccaa accgttattc attcgtgatt gcgcctgagc

11411 gagacgaaat acgcgatcgc tgttaaaagg acaattacaa acaggaatcg aatgcaaccg gcgcagggac

11481 actgccagcg catcaacaat attttcacct gaatcaggat attcttctaa tacctggaat gctgtttttc

11551 cggggatcgc agtggtgagt aaccatgcat catcaggagt acggataaaa tgcttgatgg tcggaagagg

11621 cataaattcc gtcagccagt ttagtctgac catctcatct gtaacatcat tggcaacgct acctttgcca

11691 tgtttcagaa acaactctgg cgcatcgggc ttcccataca agcgatagat tgtcgcacct gattgcccga

11761 cattatcgcg agcccattta tacccatata aatcagcatc catgttggaa tttaatcgcg gcctcgacgt

11831 ttcccgttga atatggctca taacacccct tgtattactg tttatgtaag cagacagttt tattgttcat

11901 gatgatatat ttttatcttg tgcaatgtaa catcagagat tttgagacac gggccagagc tgcagtttga

11971 tcccgagggg aaccctgtgg ttgacatgca catacaaatg gacgaacgga taaacctttt cacgcccttt

12041 taaatatccg ttattctaat aaacgctctt ttctcttagg tttacccgcc aatatatcct gtcaaacact

12111 gatagtttgt aggcccttaa ggccatgaaa ctgaaggcgg gaaacgacaa tctgatccaa gctcaagcta

12181 agctcacgtg acggaatta
